# Quantifying Reductions in *Plasmodium falciparum* Infectivity to Mosquitos: A Sample Size Calculator to Inform Clinical Trials on Transmission-Reducing Interventions

**DOI:** 10.3389/fimmu.2022.899615

**Published:** 2022-06-03

**Authors:** Jordache Ramjith, Manon Alkema, John Bradley, Alassane Dicko, Chris Drakeley, Will Stone, Teun Bousema

**Affiliations:** ^1^Radboud Institute for Molecular Life Sciences, Department of Medical Microbiology, Radboud Center for Infectious Diseases, Radboud University Medical Center, Nijmegen, Netherlands; ^2^Department for Health Evidence, Biostatistics Research Group, Radboud Institute for Health Sciences, Radboud University Medical Center, Nijmegen, Netherlands; ^3^Medical Research Council (MRC) International Statistics and Epidemiology Group, London School of Hygiene and Tropical Medicine, London, United Kingdom; ^4^Malaria Research and Training Centre, Faculty of Pharmacy and Faculty of Medicine and Dentistry, University of Science, Techniques and Technologies of Bamako, Bamako, Mali

**Keywords:** malaria, transmission, gametocyte, anopheles, mosquito, elimination, trial, oocyst

## Abstract

Malaria transmission depends on the presence of mature *Plasmodium* transmission stages (gametocytes) that may render blood-feeding *Anopheles* mosquitos infectious. Transmission-blocking antimalarial drugs and vaccines can prevent transmission by reducing gametocyte densities or infectivity to mosquitos. Mosquito infection outcomes are thereby informative biological endpoints of clinical trials with transmission blocking interventions. Nevertheless, trials are often primarily designed to determine intervention safety; transmission blocking efficacy is difficult to incorporate in sample size considerations due to variation in infection outcomes and considerable inter-study variation. Here, we use clinical trial data from studies in malaria naive and naturally exposed study participants to present an online sample size calculator tool. This sample size calculator allows studies to be powered to detect reductions in the proportion of infected mosquitos or infection burden (oocyst density) in mosquitos. The utility of this online tool is illustrated using trial data with transmission blocking malaria drugs.

## Introduction

Despite considerable improvements in access to efficacious antimalarial treatment and increased uptake of preventive strategies such as insecticide treated bed nets and indoor residual spraying, malaria is still responsible for over 2 million infections and approximately 627.000 deaths each year (1). The spread of resistance against antimalarial drugs (1) further highlights the need for additional tools in the fight against malaria. Tools that reduce the efficient transmission of malaria are considered particularly useful (2). Malaria transmission to mosquitos is initiated in the human host, when a small proportion of asexual parasites differentiate into gametocytes, the sexual reproductive forms of the parasite. When the human host is bitten by a female *Anopheles* mosquito and gametocytes are taken up with the bloodmeal, gametes are formed. Sexual reproduction starts when male gametes fertilize female gametes to form zygotes that transform into motile ookinetes that penetrate the mosquito midgut wall to form an oocyst. The presence of oocysts is typically used as evidence for successful transmission to mosquitos. After approximately 8-12 days, sporozoites are released from the oocyst and colonize the salivary glands of the mosquito, thereby rendering it infectious upon its next bite.

Transmission blocking drugs can clear or sterilize gametocytes (3–5); transmission blocking vaccines are typically designed to elicit antibodies against surface antigens of *Plasmodium* gamete [e.g. Pfs230, Pfs48/45 (6)], zygote or ookinete forms [Pfs25 (7)] or mosquito midgut antigens [AnAPN1 (8)] and thereby prevent parasite development in mosquitos. Recently, monoclonal antibodies against gamete antigens have also been proposed as transmission-reducing tools by passive immunization (9, 10).

The ultimate public health endpoint of these transmission blocking interventions is a reduction in the force of infection and thereby the incidence of malaria infection in a population (11). However, studies with these public health endpoints are complex in design, expensive and logistically challenging to implement, typically involving cluster-randomized or stepped wedge designs. Early phase testing of transmission blocking efficacy requires biological endpoints that more directly estimate of human-to-mosquito transmission. These early trials may involve naturally infected gametocyte carriers or individuals participating in controlled human malaria infection (CHMI) studies where gametocytes are induced (12–14). In both studies, mosquitos may be allowed to feed directly on the skin of parasite carriers or on a venous blood sample that is offered through a membrane (15); mosquitos can subsequently be assessed for infection status.

These functional assays allow samples from early phase clinical studies to be used for meaningful assessments of vaccine efficacy. Although there is considerable recent interest in the transition from oocyst to sporozoites, and whether this involves a developmental bottleneck (16–18), the majority of oocyst-infected mosquitos will become sporozoite-positive mosquitos (19) and until a minimum oocyst or sporozoite density is defined to render an infected mosquito infectious, the proportion of mosquitos that become infected is considered the most relevant measure of the transmissibility of naturally acquired infections. The transmission-blocking activity (TBA) of an intervention are defined as its ability to reduce the proportion of mosquitos that is infected. It is also possible that interventions do not completely prevent mosquito infection but reduce the infection burden in mosquitos (i.e. oocyst density). Transmission-reducing activity (TRA) is defined as the achieved reduction in oocyst density compared to controls (20). While studies in gametocyte carriers are typically designed to measure TBA, experiments that determine the ability of test samples to reduce the transmission of *in vitro* cultured gametocytes typically measure TRA. In standard membrane feeding assays (SMFA), high densities of cultured *P. falciparum* gametocytes are offered to mosquitos in the presence of test and control samples. SMFA are optimized to achieve high oocyst densities in control mosquitos to maximize precision and reproducibility (21). Because of this high infection intensity in control mosquitos, even highly potent samples may not prevent oocyst formation completely and TRA is the common readout of SMFA (20).

Sample size estimates for transmission blocking efficacy outcomes are challenging. When candidate vaccines, drugs or monoclonal antibodies enter clinical testing, they are typically primarily evaluated for safety in small first-in-human trials (7) that are powered on outcomes other than efficacy outcomes. Transmission assays are inherently noisy and considerable between-site variation exists in the performance of mosquito assays. Two additional complicating factors are the negative binomial distribution of oocysts (22–24), which is especially relevant when using oocyst density as an outcome measure, and the strong correlation between mosquito observations from the same individual in studies with naturally infected gametocyte carriers. Despite these challenges, mosquito feeding assays offer opportunities to maximize informativeness of trials with transmission-blocking interventions. In this context, we describe a negative binomial mixed effects model for TRA endpoints and a mixed effects logistic regression for TBA endpoints. Using these models, we designed a calculator tool that allows i) power analysis for transmission blocking intervention trials based on both TRA or TBA efficacy endpoints by means of mosquito feeding assays, ii) statistical analysis of data to either determine reference values for the power analysis or to quantify TRA and TBA as study outcomes. The practical application of the calculator is demonstrated with two clinical datasets.

## Materials and Methods

### The Statistical Models

In order to calculate empirical power to detect reduction in oocyst prevalence/proportion of infected mosquitos, we simulated transmission data to estimate the effects of different levels of Transmission Blocking Activity (TBA). These data contain a binary outcome (a mosquito can either be infected or not infected), therefore a mixed effects logistic regression model was used. For power calculations for studies with reduction of oocyst density as an endpoint, the negative binomial distribution of oocysts has to be taken into account. This distribution is required as the majority of oocysts is found in a small proportion of all mosquitos (22–24). Therefore, to calculate the empirical power to estimate the effect of different levels of Transmission Reducing Activity (TRA), a mixed effects negative binomial regression model was used. For both models, we used mixed effects models, meaning we modelled both fixed effects of TBA and TRA as well as random intercepts. Both logistic regression and negative binomial regression models assume that the data are independent. Violations in this assumption leads to underestimated standard errors and thus an increased likelihood of false positive findings. Including random intercepts in the models, allows for the correlation between outcomes for mosquito samples from the same participant to be accounted for; which we refer to as the intra-cluster correlation. The random intercepts are used to allow participant-level variation in pre-intervention transmissibility, i.e. a participant-specific baseline proportion of infected mosquitos (for TBA models) or participant-specific baseline geometric mean oocyst density (for TRA models).The mathematical details for the data simulation algorithm and the statistical models and tests are given in the **Supplementary Information**.

**Figure 1:**
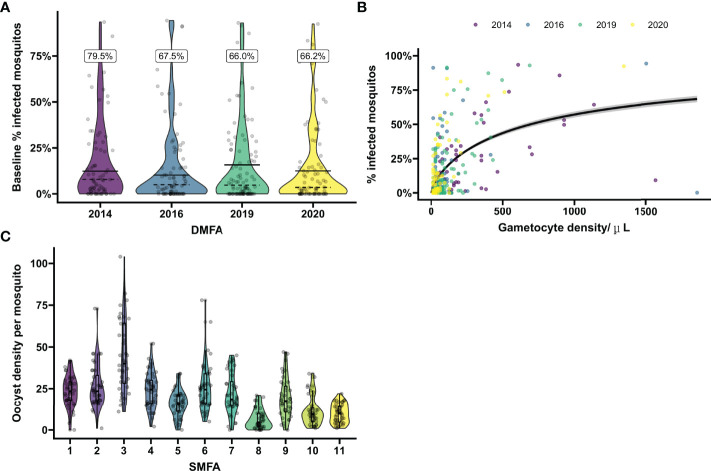
Baseline mosquito infectivity in studies assessing transmission blocking or transmission reducing activity. **(A)** Variation in the proportion of infected mosquitos from natural gametocyte carriers. Violin plots show the percentage of infected mosquitos at baseline as determined by mosquito feeding assays on microscopically detected *P. falciparum* gametocyte carriers in Mali prior to intervention in four separate transmission blocking intervention trials performed in 2014, 2016, 2019 and 2020. On average 66.2 mosquitos were dissected per sample. Dashed lines indicate the mean baseline percentage of infected mosquitos when including all enrolled gametocyte carriers. The black lines indicate the mean baseline percentage of infected mosquitos including only samples that were infectious to mosquitos. The percentage above the plot gives the percentage of infectious gametocyte carries for each year. Dots show the values of individual baseline samples. **(B)** Microscopically estimated gametocyte density in relation to mosquito infection rates. The relationship between log gametocyte density by microscopy and probability of a mosquito being infected was modelled with logistic regression. The black line indicates the expected proportion of infected mosquitos across given gametocyte densities. Samples positive for gametocytes by microscopy at baseline were included from the four trials presented in **(A)**. To demonstrate the effect of entry criteria (i.e. the minimum gametocyte density required for participation) on pre-intervention infectiousness, the average slope over the total of 4 trials was presented. However, average gametocyte density as well as the modeled slopes differ between the different years, emphasizing the variability of baseline proportion of infected mosquitos and need of site-specific baseline parameters for sample size calculations. Dots show values per individual sample, colors correspond with the trials as presented in **(A)**. **(C)** Variation in oocyst density in experiments with *in vitro* cultured gametocytes. Violin plots show the oocyst density per mosquito for pooled malaria-naïve control sera in 11 separate mosquito membrane feeding experiments with cultured *P. falciparum* NF54 gametocytes. Sera were tested in duplicate with 20 mosquito dissections per sample. Boxes indicate IQR and median oocyst density per experiment, whiskers indicate full range. Dots indicate oocyst counts in individual mosquitos.

To estimate empirical power for TBA, the calculator relies on user specifications of i) baseline proportion infected mosquitos, ii) anticipated TBA and iii) the intra-cluster correlation that is used to directly estimate the variance of the random effects. The value that is entered for baseline proportion of infected mosquitos is ideally based on site-specific data from preceding (pilot) studies or, if unavailable, on best estimates from existing literature, taking into account variation in gametocyte density in the study population (**Figure 1B**). The percentage of transmission inhibition that we expect the studied intervention to achieve, the anticipated TBA, can be estimated based on pre-clinical data. The intra-cluster correlation reflects the correlation of infection between mosquitos fed on the same participant and is determined based on the variance of the intercepts in the pilot dataset. Thus likelihood of infection in mosquitos fed from one sample is highly correlated when the intra-cluster correlation is close to 1, and independent when the intra-cluster correlation is 0.

Similarly, to estimate empirical power for TRA, the calculator relies on four user-defined specifications of: i) baseline geometric mean oocyst density, ii) anticipated TRA, iii) variance of the random intercepts, and iv) the dispersion parameter. The baseline geometric mean oocyst density, variance of the random effects and dispersion parameter can be calculated in the data analysis tool preferably using site-specific individual mosquito level data from preceding (pilot) studies. The anticipated TRA is user-defined and can for example be based on pre-clinical data. The dispersion parameter in the negative binomial regression model controls over-dispersion – which is the case when the empirical variation in the data is larger than that predicted from the model. The dispersion parameter and random effects variance together control the extent of inter-cluster correlation, for which an estimate is displayed in the input panel of the power calculator.

Finally, for both power calculations, a user-specified level of significance and a testing threshold should be specified. For the levels of significance, one of two choices is possible, 0.025 or 0.05, of which 0.025 is often preferred for one-sided tests/superiority trials and applicable for most envisaged use scenarios where TBA or TRA is anticipated to exceed a certain threshold value. This threshold can be zero, when merely testing whether an intervention reduces transmission compared to the pre-intervention control condition; often it is more informative to demonstrate that TBA/TRA is significantly larger than a higher threshold, for instance a minimum TRA of 80% has historically been proposed to identify potent interventions for further development. It is relevant to realize that this threshold TBA or TRA value is different from the anticipated TBA/TRA value, the value that we expect and is typically higher than the threshold level we aim to compare it to.

### Study Populations and Reference Values

In order to provide reference values to inform envisioned future transmission studies, two datasets from recent clinical trials with transmission endpoints were analyzed in the calculator. For oocyst prevalence data (i.e. the proportion of infected mosquitos), individual-level data from studies examining the impact of transmission-reducing antimalarial drugs were used (25). This exemplar dataset was selected to illustrate reductions in the proportion infected mosquitos (i.e. transmission blocking activity; TBA) following interventions. In this study, naturally infected gametocyte carriers were included and transmissibility to mosquitos was assessed before and after a drug intervention. Briefly, naturally infected gametocyte carriers with microscopically detectable *P. falciparum* gametocyte densities (>16 gametocytes/µL) were recruited and treated with conventional artemisinin-combination therapy (a 3-day regimen administered by weight of 320mg dihydroartemisinin and 40 mg piperaquine per tablet) alone or combined with low doses of gametocytocidal drugs (0.0625 mg/kg, 0.125mg/kg, 0.25 mg/kg 0.5 mg/kg of primaquine). Before and after initiation of treatment, venous blood was drawn and offered to locally reared mosquitos that were examined 7 days later for the presence of oocysts (binary outcome: absent/present) with on average 70.5 mosquitos dissected per blood sample. This key dataset was complemented with data of four independent trials (4, 25–27) that evaluated the transmission blocking efficacy of gametocytocidal drugs by means of direct membrane feeding assays in a single site in Ouelessebougou, Mali. These additional data were used to examine variation in the proportion of infected mosquitos at baseline, prior to administration of any transmission blocking drugs. The number of gametocytes determined by microscopy at screening was used to demonstrate how parasitological enrolment criteria such as gametocyte density influenced baseline infectivity and thereby the efficiency of TBA assessments.

For oocyst density data, we used standard membrane feeding assay (SMFA) results from a clinical trial NCT04238689 with a highly potent transmission blocking monoclonal antibody as reference dataset. The efficacy of the monoclonal antibody was studied in malaria-naïve study participants; their serum samples being offered to mosquitos in the presence of high densities of cultured *P. falciparum* NF54 gametocytes in the SMFA, rendering this reference appropriate to illustrate reductions in oocyst density as an outcome (i.e. transmission-reducing activity; TRA). This dataset included 20 subjects before administration of a transmission blocking monoclonal antibody and 10 subjects post administration who were selected to have partial TRA. On average 19.4 mosquitos were dissected with a median oocyst density of 42 in mosquitos fed on pre-intervention samples. Post-administration samples were selected where the mean TRA value was approximately 80% (median 77.1%, range 62.4-86.1%), a TRA value traditionally used as threshold to support further development of transmission-blocking interventions (28, 29). This dataset was complemented with 11 independent experiments performed for the abovementioned clinical trial (30) to examine variance in oocyst density for SMFA experiments conducted at the same site (Radboudumc, Nijmegen, the Netherlands). The only entry criterion for SMFA experiments was a proportion of infected mosquitos of >70%, a pre-defined quality control threshold (20).

All trials in Mali received ethics approval by the Ethics Committee of the Faculty of Medicine, Pharmacy, and Dentistry of the University of Science, Techniques, and Technologies of Bamako (Bamako, Mali), and the Research Ethics Committee of the London School of Hygiene & Tropical Medicine (London, UK). The trial in the Netherlands received approval from the Arnhem-Nijmegen Committee on Research Involving Humans.

### Simulation Scenarios

To show a range of power calculations for TRA and TBA respectively, we allowed different combinations of parameter values for the data simulation and empirical power calculations. For both TBA/TRA we allowed sample sizes *n* = (10, 20, 30, 40) for participants and *m* = (20, 40, 60) for mosquitos, based on conventional group sizes (31–35). We used consensus thresholds of meaningful efficacy (24, 28, 29, 36) to define anticipated *TBA*/*TRA* = (70%, 80%, 95%) and thresholds for detecting *TBA*/*TRA* larger than *τ* = (50%, 80%, 90%). Further, for *TRA* we considered baseline geometric mean oocyst densities of *μ*_0_ = (20, 30, 45) (37) and for *TBA* we considered baseline proportion infected mosquitos of *p*_0_ = (10%, 15%, 25%) based on a meta-analysis of membrane feeding experiments (38). For intra-cluster correlation we considered values of 0 for independence and 0.5 or 0.35 for TBA or TRA analyses, respectively, as motivated by the reference data. For TRA, the intra-cluster correlation depends on both variance of the random effects and the dispersion parameter, so to keep the results comparable, we used a fixed dispersion parameter as estimated by the data analysis and varied the variance of the random effects to determine the intra-cluster correlation (0, 0.35). We only considered a significance level of 0.025, being interested in one-tailed testing whether TRA or TBA were larger than a pre-defined threshold. A significance level of 0.025 for a one-tailed test is essentially the equivalent of a significance level of 0.05 for a two-tailed test; the empirical power for a significance level of 0.05 is always larger.

### Software

All data analysis was conducted using R4.1.1 (39). in RStudio (40) making use of the mgcv package (41, 42) for the analysis. Rshiny was used to develop the app (43). The app is currently hosted on https://bousema-lab.shinyapps.io/transmission_sample_size/.

## Results

**Figure 2:**
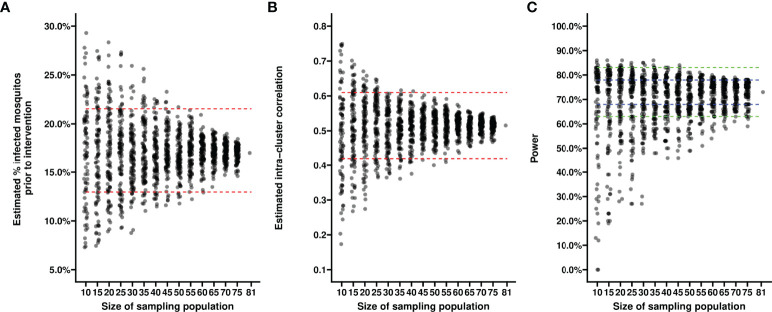
Estimates of baseline proportion of infected mosquitos using different sample sizes. Each dot reflects the estimated average percentage of infected mosquitos (y-axis) **(A)** or the estimated intra-cluster correlation **(B)** in a random sample of participants from a transmission blocking intervention trial performed in Mali in 2014. Each dot in **(C)** reflects the power estimate derived from the estimates of average percentage of infected mosquitos and intra-cluster correlation from a random sample of participants, based on an anticipated TBA of 90%, a threshold TBA of 80%, a number of participants of 20, a number of dissected mosquitos of 30 and a significance level of 0.025. Sizes of the sampled populations range from 10 to 75 participants, with intervals of 5 participants (x-axis). For each sample size scenario, 100 random samples selected without replacement were taken from the trial dataset with a total of 81 participants. Red dashed lines indicate 95% confidence intervals of the averages in the total trial population. Blue dashed lines indicate a 5% margin of error, green dashed lines indicate a 10% margin of error from the power estimate based on the reference values derived from the complete dataset.

First, we evaluated the variance in baseline proportion of infected mosquitos from four previous transmission blocking intervention trials. Mosquito infection prevalence prior to the intervention differed per study and varied from 14.2% – 17.4% when including non-infectious participants, or from 21.1% - 24.0% when including only infectious participants (**Figure 1A**). The proportion of infected mosquitos at baseline was strongly correlated with concurrent gametocyte density (**Figure 1B**). Whilst this association has repeatedly been described (44–47) and the current analysis was not intended to improve on such estimates, the association is of immediate relevance in designing studies and selecting the study population. The proportion infected mosquitos at baseline is highly dependent on eligibility criteria: e.g. when using a threshold of >50 gametocytes/µL as selection criterion, the average proportion of infected mosquitos at baseline was 25%, increasing to 35% when using a threshold of >100 gametocytes/µL, and 44% for a threshold of >200 gametocytes/µL. The shape of the association between gametocyte density and mosquito infection rates may vary between study sites and years (48). To inform the size of pilot experiments to determine site-specific baseline data on the proportion of infected mosquitos, we explored how precision in estimates of baseline infectivity and intra-cluster correlation depends on the size of the study population. For this, we used one study population [2014 study in **Figure 1A** (25)] to randomly select subjects from. The true proportion of infected mosquitos for the average participant in the entire population (n=81) was 17.0% (95% CI: 13.0%, 21.6%); the true intra-cluster correlation in the entire population was 0.52 (95% CI: 0.42, 0.61). The distribution of estimates of the baseline infectivity and the intra-cluster correlation for 100 simulations with sampling sizes of 10 to 75 participants are presented in **Figures 2A, B**, respectively. When taking 100 random samples of 40 participants, 84% of the estimations of baseline proportion of infected mosquitos were within the 95% CI of the complete dataset of 81 participants. With this same sampling size of 40 participants, 93% of the estimations for intra-cluster correlation were within the 95% CI of the complete dataset. Forty participants in pilot experiments may thus provide reasonably precise estimates of baseline parameters. We next performed power calculations using an anticipated TBA = 90%, a threshold TBA of 80%, number of participants = 20 and number of dissected mosquitos per sample = 30 and a significance level of 0.025. The power was 73% using the true reference parameters of the full dataset (i.e. baseline proportion of infected mosquitos = 17.0% and intra-cluster correlation = 0.52). For a sample size of 40, approximately half of the estimates for proportion infected and intra-cluster correlation led to an estimated power within the acceptable power range with 5% margin of error, and approximately 80% were within the acceptable power range when the margin of error was increased to 10% (**Figure 2C**).

Similarly, variance in baseline oocyst density was evaluated using the SMFA outcomes of malaria-naïve control sera from 11 separate experiments. Despite experiments being conducted with the same parasite line (NF54) and mosquito species (*An. stephensi*) in the same laboratory, the mean oocyst density per mosquito was highly variable over the experiments and reached 6.5 – 44.6 oocysts/mosquito (**Figure 1C**). These findings illustrate the need for site-specific baseline parameters for sample size calculations and, for SMFA, adequate controls.

**Figure 3:**
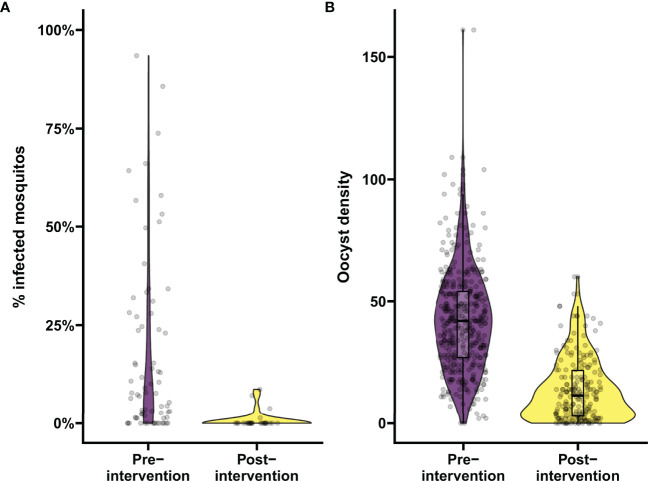
Analyzing the proportion of infected mosquitos and oocyst burden in mosquitos using the app. Demonstration of the data analysis output from the app. **(A)** Analysis of changes in percentage of infected mosquitos. Violin plots show the percentage of infected mosquitos pre- and post-intervention as determined by mosquito feeding assays from natural gametocyte carriers in a transmission trial performed in Mali in 2014, in purple and yellow respectively. On average 70.5 mosquitos were dissected per sample. **(B)** Analysis of changes in infection burden (oocyst density). Violin plots show oocyst density pre- and post-intervention from samples selected from a trial evaluating a transmission blocking monoclonal antibody in malaria naïve subjects by means of mosquito feeding experiments with cultured gametocytes, in purple and yellow respectively. On average 19.4 mosquitos were dissected per sample. Boxes indicate median and IQR, whiskers indicate range, outliers are presented as dots and were defined as >1.5*IQR.

To demonstrate the utility of the tool for data analysis, data from a previous transmission blocking intervention trial using proportion of infected mosquitos by membrane feeding as the primary outcome measure (25) were analyzed in the mixed-effects logistic regression model. For a trial with natural gametocyte carriers, the reduction in the proportion of infected mosquitos (TBA) is the preferred outcome. TBA was estimated as a function of the baseline proportion of infected mosquitos and the estimated odds ratio from the model. These estimates included individuals who were not infectious to mosquitos. Prior to treatment, 18.5% (15/81) of the participants in this trial was non-infectious (25) and, including these individuals, the estimated proportion of infected mosquitos for the average person prior to treatment was 17.0% (95% CI: 13.0%, 21.6%) (2014 study in **Figure 1A**). Based on previous demonstrations of the potency of primaquine in preventing transmission (25, 49), we used the analysis tool to test whether transmission was reduced by at least 80% following primaquine treatment. In our exemplar dataset, the proportion of infected mosquitos was reduced to 2.0% (95% CI: 1.2%, 3.2%) (**Figure 3A**). TBA was estimated at 88% (95% CI: 82.2%, 91.9%), significantly larger than the threshold of 80% (p=0.0058). The intra-cluster correlation was estimated to be 0.52.

Subsequently, we used the data analysis tool to compare two out of five intervention arms from the same trial. In the study arm receiving 0.125mg/kg of primaquine, the estimated proportion of infected mosquitos for the average person was 11.0% prior treatment, which was reduced to 1.6% after intervention. The TBA is estimated to be 85.88% (95% CI: 76.93%, 91.36%), not significantly higher than the threshold of 80% (p=0.0824). For the study arm receiving 0.5 mg/kg of primaquine, the estimated 12.7% of mosquitos that was infected for the average person at baseline, was reduced to 0.8% post-intervention, resulting in a TBA of 94.0% (95% CI: 86.4%, 97.4%), significantly higher than the threshold of 80% (p=0.0021). TBA was not statistically significantly different between arms (p=0.0793). The intra-cluster correlation was estimated to be 0.47.

**Figure 4:**
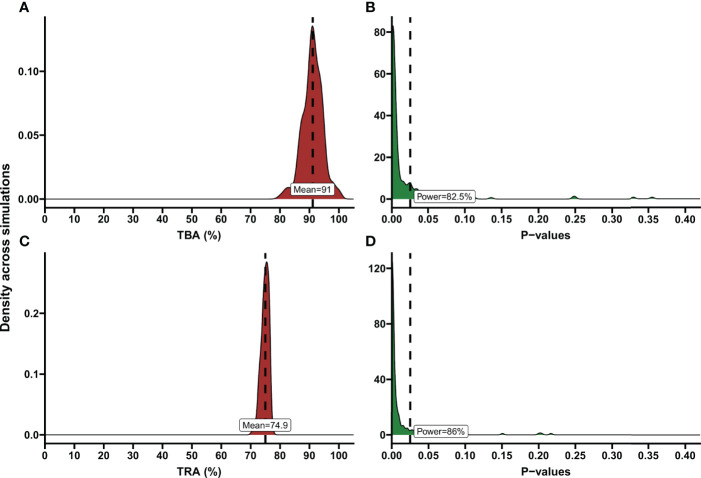
Examples of TBA and TRA power estimations. Demonstration of power calculation output of the app. These are illustrated using density plots, where the y-axis indicates the kernel density estimates for the values on the x-axis, which is a smoothed version of the histogram. **(A)** Percentage of transmission blocking activity (TBA) across simulations. The dashed line shows the mean TBA across stimulations; the red shaded area shows the distribution of TBA estimates in simulations. As an example, variables based on the data analysis of a transmission blocking intervention trial performed in Mali, 2014 were entered in the power calculator. Number of simulations: 200, Anticipated TBA: 90%, Threshold: 80%, Number of subjects: 20, Number of dissections per subject: 40, Baseline proportion infected: 17.02%, Intra-cluster correlation: 0.5, Level of significance: 0.025. **(B)** P-values across simulations, using the variables as described in **(A)**. Dashed line shows level of significance (0.025); the green shaded area shows the distribution of p-values across simulations. Estimated power is presented in the text box. **(C)** Percentage of transmission reducing activity (TRA) across simulations. Dashed line shows the mean TRA across stimulations. As an example, variables based on the data analysis of a trial evaluating the TRA of a monoclonal antibody were entered in the power calculator. Number of simulations: 200, Anticipated TRA: 75%, Threshold: 70%, Number of subjects: 10, Number of dissections per subject: 20, Geometric mean number of oocysts for the average subject pre-treatment: 41.27, Anticipated standard deviation of the random intercepts: 0.393, Anticipated dispersion parameter 3.316, Intra-Cluster Correlation: 0.35, Level of significance: 0.025. **(D)** P-values across simulations, using the variables as described in **(C)**. Dashed line shows level of significance (0.025). Estimated power is presented in the text box.

Informed by these reference values we used a baseline proportion of infected mosquitos of 15% and an intra-cluster correlation of 0.5 to perform power calculations for envisioned future trials with transmission reducing interventions. Alternatively, all trials with the same enrolment criteria (**Figure 1A**) could be used to inform reference values for an envisioned future trial; these values are provided in **Supplementary Table 1**. We used several different sample sizes of human participants (n=10, n=20, n=30, n=40), numbers of mosquitos dissected (m=20, m=40, m=60), anticipated TBA values (70%, 80%, 95%) that we expect the efficacy of our studied transmission blocking intervention will be, and threshold TBA values (>50%, >80%, >90%) that we wish to show the TBA of the studied intervention exceeds. One example of the output of the power calculator for such a power estimation is shown in **Figure 4**. We repeated simulations using a range of baseline proportion of infected mosquitos (10%, 15% and 25%), and an intra-cluster correlation of 0; results are shown in **Table 1**. Note that this table is based on parameters that are representative for the study site of the datasets analyzed in this paper, but different values for intra-cluster correlation and other baseline parameters may represent better the users’ own study site and can be computed using the data analysis tool.

**Table 1:** Power for trials using reduction in proportion of infected mosquitos as functional outcome.

			*ICC* = 0									*ICC* = 0.5								
	n	m	Anticipated TBA=70			Anticipated TBA=80			Anticipated TBA=95			Anticipated TBA=70			Anticipated TBA=80			Anticipated TBA=95		
			τ =50	τ =80	τ =90	τ =50	τ =80	τ =90	τ =50	τ =80	τ >90	τ =50	τ =80	τ =90	τ =50	τ =80	τ =90	τ =50	τ =80	τ =90
*p*_0_ = 10%	10	20	17.5	0	0	30	0	0	48.5	0	0	18.5	0	0	36.5	0	0	29	1.5	0
		40	40	0.5	0	84.5	2.5	0	81	27.5	0	49	0	0	78.5	0	0	53.5	17.5	0
		60	52	0	0	89.5	4.5	0	91.5	71	0.5	65.5	0	0	91.5	0.5	0	64.5	38	1
	20	20	32	0	0	70	1.5	0	91.5	27.5	0	53	0	0	82.5	0.5	0	53.5	17.5	0
		40	66	0	0	99	2	0	96	87	14.5	86	0	0	99	3	0	76.5	65.5	4
		60	82.5	0	0	100	3	0	100	100	35	95.5	0.5	0	99.5	8	0	84.5	81	20
	30	20	30	0	0	90	0	0	100	79	0	67.5	0	0	96.5	1.5	0	78.5	60	1
		40	74	0	0	100	0	0	100	100	40.5	95.5	0	0	100	6	0	94	92	19.5
		60	92	0	0	100	0	0	100	100	48	99	0	0	100	7.5	0	97	97	52
	40	20	51	0	0	100	0	0	100	99	2.5	83.5	0	0	100	1.5	0	87	84	2
		40	99.5	0	0	100	0	0	100	100	66.5	100	0	0	100	7.5	0	96	96	50.5
		60	98.5	0	0	100	0	0	100	100	79.5	100	0	0	100	10	0	99	99	78
*p*_0_ = 15%	10	20	29	0	0	55	1	0	73.5	2.5	0	35	0	0	61	0.5	0	48	9	0
		40	58	0	0	92	5	0	94	63	0	65.5	0	0	90	1	0	72	46.5	1
		60	65	0	0	99.5	3.5	0	99	90	19.5	77.5	0	0	99	2.5	0	79.5	70	6.5
	20	20	42.5	0	0	89.5	7	0	96	61	0	73	0	0	95.5	3	0	70	55	3
		40	87.5	0	0	100	5	0	100	99	22	93.5	0	0	100	7	0	88	87	26
		60	92.5	0	0	100	6	0	100	100	40	99.5	0	0	100	10.5	0	93.5	93.5	53.5
	30	20	57.5	0	0	97.5	0	0	100	83	10.5	77	0	0	99.5	2	0	91	88.5	7.5
		40	98	0	0	100	1	0	100	100	37	100	0	0	100	9	0	97	97	56
		60	100	0	0	100	3.5	0	100	100	57	100	0	0	100	12.5	0	98	98	78
	40	20	71	0	0	100	0	0	100	96.5	23	91	0	0	100	2.5	0	96	95.5	21
		40	100	0	0	100	0	0	100	100	56.5	100	0	0	100	9.5	0	99	99	79
		60	100	0	0	100	0	0	100	100	82.5	100	0	0	100	14	0	100	100	92.5
*p*_0_ = 25%	10	20	39	0	0	87	3.5	0	95	55	0	51.5	0	0	91	1	0	71	35.5	0.5
		40	78.5	0	0	100	2.5	0	100	96.5	26	81.5	0	0	99.5	4.5	0	88.5	84	12.5
		60	88.5	0	0	100	7	0	100	100	41	88.5	0	0	100	7	0	90	89	32
	20	20	59	0	0	100	6.5	0	100	94	11.5	84	0	0	100	5	0	91.5	89.5	14.5
		40	94	0	0	100	5.5	0	100	100	40.5	96	0	0	100	10	0	97.5	97.5	63
		60	100	0	0	100	11.5	0	100	100	70.5	99	0	0	100	12	0	98.5	98.5	76.5
	30	20	80	0	0	100	0	0	100	100	18	94	0	0	100	3.5	0	98	98	40.5
		40	100	0	0	100	0	0	100	100	80.5	100	0	0	100	8	0	100	100	81
		60	100	0	0	100	1.5	0	100	100	92.5	100	0	0	100	12.5	0	100	100	87.5
	40	20	92	0	0	100	0	0	100	100	19	98.5	0	0	100	3	0	100	100	55
		40	100	0	0	100	0	0	100	100	97	100	0	0	100	10.5	0	100	100	93.5
		60	100	0	0	100	0	0	100	100	99	100	0	0	100	16.5	0	100	100	97.5

ICC, intra-cluster correlation; TBA, transmission blocking activity; n, number of participants; m, number of mosquitos dissected; p_0_ baseline proportion of infected mosquitos; τ, the threshold at which we wish to test that the anticipated TBA exceeds.

Similarly, we analyzed data of a recent trial using reductions in oocyst density (i.e. transmission reducing activity; TRA) by SMFA as the endpoint (30) in the mixed-effects negative binomial regression model. The number of 41.3 oocysts per mosquito for the average person at baseline was reduced to 10.5 after intervention (**Figure 3B**), resulting in a TRA of 74.5% (95% CI: 71.3, 77.3), significantly higher than the threshold of 70% (p=0.0035). The standard deviation of the random intercepts was 0.393 and the dispersion parameter was estimated as 3.316 which were used to estimate an ICC of 0.35.

Next, we compared two of the intervention arms from the same trial in the analysis tool. For the first arm the number of 52.7 oocysts per mosquito for the average person at baseline was reduced to 20.2 after intervention, resulting in a TRA of 61.6% (95% CI: 55.0, 67.3), not higher than the threshold of 70% is (p=0.9987). For the second arm, the number of 35.9 oocysts per mosquito for the average person at baseline was reduced to 5.3 oocysts per mosquito after intervention, resulting in a TRA of 85.3%, significantly higher than the threshold of 70% (p<0.001). Estimates of TRA were significantly different between arms (p<0.001). The standard deviation of the random intercepts was 0.36 and the dispersion parameter was estimated as 3.358 which were used to estimate an ICC = 0.31.

Power calculations for envisioned future studies were performed using a number of 20, 30 or 45 oocysts per mosquito for the average person at baseline and an intra-cluster correlation of 0 or 0.35 using several variations in trial characteristics (number of participants, number of dissections per sample, TRA threshold, anticipated TRA) (**Table 2**). An example of the power calculator output is shown in **Figure 4**. The following general patterns can be derived and are in agreement with the literature (21): i) for both TBA and TRA, empirical power is highly dependent on site specific parameters: oocyst density or proportion of infected mosquitos at baseline and intra-cluster correlation; ii) power can be increased substantially by using higher number of dissected mosquitos per sample.

**Table 2:** Power for trials using reduction in oocyst density as functional outcome.

			*ICC*= 0									*ICC*= 35								
	n	m	Anticipated TRA=50			Anticipated TRA=70			Anticipated TRA=80			Anticipated TRA=50			Anticipated TRA=70			Anticipated TRA=80		
			τ =35	τ =40	τ =45	τ =55	τ =60	τ =65	τ =65	τ =70	τ =75	τ =35	τ =40	τ =45	τ =55	τ =60	τ =65	τ =65	τ =70	τ =75
*μ*_0_ = 20	5	10	60.5	32	13.5	89	59.5	24.5	99	85.5	43.5	55.5	32.5	14.5	87.5	60	21.5	99.5	86	42
		20	87.5	61	17.5	99.5	92	36.5	100	99	69	87	56.5	16	99.5	89.5	33	100	98	67.5
		30	97.5	78	24	100	98	58.5	100	100	89.5	97	77.5	25	100	97.5	59.5	100	100	91
	10	10	91.5	57	14.5	100	93	39.5	100	99.5	70	88.5	57.5	16.5	99.5	91	37.5	100	99	67
		20	100	83	41	100	100	71	100	100	93	99	86	36	100	100	71	100	100	91
		30	100	95.5	49	100	100	82.5	100	100	99.5	100	96	48	100	100	83.5	100	100	100
	20	10	100	94	26.5	100	100	70.5	100	100	96.5	100	92	31.5	100	100	73.5	100	100	94
		20	100	100	66	100	100	97	100	100	100	100	99.5	63.5	100	100	90	100	100	100
		30	100	100	74.5	100	100	97.5	100	100	100	100	100	72.5	100	100	99	100	100	100
*μ*_0_ = 30	5	10	58.5	32	11.5	95	63	25.5	100	87.5	44.5	59	34	11	92	65.5	20	99	89	45.5
		20	90	57	15	99.5	93	41.5	100	100	74.5	89	55.5	16.5	100	91	40	100	99	71
		30	100	81	28.5	100	99.5	57	100	100	93	97.5	81.5	27.5	100	98.5	57.5	100	100	92
	10	10	91	59	17	100	95	45	100	100	76	92.5	58.5	14.5	100	93.5	39.5	100	99.5	76
		20	99.5	86.5	32.5	100	100	74	100	100	95	99.5	86	32.5	100	100	72	100	100	93
		30	100	97	52	100	100	84	100	100	100	100	97.5	50.5	100	100	85.5	100	100	99.5
	20	10	100	90.5	36	100	100	80	100	100	95.5	100	92.5	33	100	100	78	100	100	97.5
		20	100	98	56.5	100	100	93.5	100	100	100	100	100	63	100	100	93	100	100	100
		30	100	100	76	100	100	100	100	100	100	100	100	73.5	100	100	99	100	100	100
*μ*_0_ = 45	5	10	61	38	10	95.5	68.5	29	100	95	45.5	59.5	34	10	95	67.5	28.5	100	93.5	43.5
		20	90	58	19	100	94.5	39.5	100	100	77	90.5	60.5	18.5	100	92	37.5	100	100	74.5
		30	98.5	81	27	100	99.5	65	100	100	93.5	98.5	83.5	24	100	99.5	63	100	100	94
	10	10	91	59.5	20.5	100	96.5	49	100	100	78.5	91	60.5	15.5	100	97	45.5	100	100	76
		20	100	87	36	100	100	71.5	100	100	94.5	99.5	89.5	37	100	100	73.5	100	100	95
		30	100	98.5	49.5	100	100	86	100	100	100	100	99	49	100	100	89.5	100	100	100
	20	10	100	93.5	38	100	100	82.5	100	100	100	100	91	33	100	100	80	100	100	99
		20	100	100	64.5	100	100	97.5	100	100	100	100	100	65.5	100	100	95	100	100	100
		30	100	100	76	100	100	100	100	100	100	100	100	79	100	100	99.5	100	100	100

ICC, intra-cluster correlation; TBA, transmission blocking activity; n, number of participants; m, number of mosquitos dissected; μ_0_ baseline oocyst density; τ, the threshold at which we wish to test that the anticipated TBA exceeds.

## Discussion

We present a mathematical framework to calculate power and analyze data in transmission blocking intervention studies using either TBA or TRA as the efficacy outcome. These methods are made accessible in an online tool that allows users to perform their own analyses and power calculations in pre- and post-intervention comparisons as well as study designs that compare an intervention- to a control group.

After years of relative neglect, there is an increasing interest in the impact of novel antimalarial drugs on transmission (50–52) and transmission blocking vaccines (53). An important advantage of transmission-blocking interventions is that there are informative biological endpoints for efficacy (11) for which there have been efforts to qualify assays (54). However, the large number of variables in mosquito feeding assays complicates both analysis and power calculations for transmission blocking intervention studies. The anticipated transmission inhibition, number of participants, number of mosquitos, baseline gametocyte density, baseline proportion of infected mosquitos or oocyst density all affect the power in such trials. What adds to that complexity, is that there are site-specific conditions (38) such as feeding protocol (e.g. direct skin versus membrane feeding, duration of feeding, type of artificial membrane), donor characteristics (e.g. minimum parasite or gametocyte density) and mosquito characteristics (e.g. receptivity and survival rate), that lead to site-specific differences in baseline oocyst density, proportion of infected mosquitos or number of mosquitos available for dissection (38, 48). As illustrated in the current analysis, there can even be considerable variation between experiments conducted at the same study site with the same procedures and inclusion criteria (**Figure 1**) (48, 55). It is thus imperative to obtain site-specific baseline estimates of infectivity in pilot experiments prior to designing transmission-blocking intervention trials. For studies in natural gametocyte carriers, one approach is to determine in pilot experiments what percentage infected mosquitos can be achieved with the enrolment criteria of the envisaged clinical trial. We explored the number of gametocyte carriers that should be included in such pilot experiments by randomly selecting participants from our study population. Including a minimum of 40 donors allowed us to approximate the ‘true’ mosquito infection prevalence and intra-cluster correlation with sufficient precision to allow power calculations. An alternative or complementing approach would be to test whether the association between gametocyte density and mosquito infection rates in the envisaged study population follows that of a recent multi-site study (48) and subsequently decide what a minimum gametocyte density should be for study participants to be enrolled in the study. Using stringent eligibility criteria can increase the pre-intervention proportion of infected mosquitos, resulting in an increased efficiency of the trial. As an example, using the reference values of the transmission blocking intervention trial described above (i.e. intra-cluster correlation = 0.52, anticipated TBA = 88%, number of participants = 20, number of dissected mosquitos per experiment = 40, threshold value of TBA = 80%), by only including participants with a minimal gametocyte density of 100 gametocytes/µL, the baseline proportion of infected mosquitos can increase from 17.0% to 34.6%, resulting in a power increase from 82.5% to 92%. Whilst it may be challenging to recruit these rare high-density gametocyte carriers, it will increase study power.

Our analyses emphasize the value of a site-adaptable tool to analyze data and make power calculations for trials with transmission-blocking interventions. The high variability in baseline data highlights that use of site-specific baseline data is strongly recommended for obtaining reference values to enter in the power calculator, instead of using the preset reference values or power estimations as presented in **Tables 1**, **2**, that are based on the datasets described in this manuscript.

An important consideration to consider when using the app is that the power calculations are based on finding TRA or TBA above a certain threshold within study arms or within a total study population, but not on the comparison of transmission blocking efficacy between two intervention arms. Although the app is not designed for this, the data analysis tool does offer the opportunity for some alternative analyses. If the number of infectious individuals (i.e. the number of individuals infecting at least one mosquito) is a preferred outcome instead of TBA or TRA, the analysis tool can be used by generating a dataset with only one fictive dissected mosquito per individual and entering 0 for non-infectious and 1 for infectious individuals in the column for number of infectious mosquitos. Additionally, the analysis tool could be used for other paired assessments of infectivity (for example the relative transmission of a primary vivax infection compared to a recrudescent infection).

In conclusion, we have developed a tool for analysis and power calculation of transmission blocking intervention trials that is accessible on https://bousema-lab.shinyapps.io/transmission_sample_size/. This supports the inclusion of functional mosquito feeding assays to assess intervention efficacy in early phase trials and thereby maximize their informativeness. This may accelerate the clinical development of transmission blocking interventions. At present, mosquito feeding assays remain a surrogate endpoint for public health impact that requires confirmation; predicting the association between intervention efficacy in terms of reductions in the proportion of infected mosquitos and the public health impact at population level is a high priority.

## Data Availability Statement

Datasets used to demonstrate the utility of the app are available upon request to the corresponding author. Requests to access these datasets should be directed to teun.bousema@radboudumc.nl.

## Ethics Statement

All trials in Mali received ethics approval by the Ethics Committee of the Faculty of Medicine, Pharmacy, and Dentistry of the University of Science, Techniques, and Technologies of Bamako (Bamako, Mali), and the Research Ethics Committee of the London School of Hygiene & Tropical Medicine (London, UK). The trial in the Netherlands received approval from the Arnhem-Nijmegen Committee on Research Involving Humans. The patients/participants provided their written informed consent to participate in this study.

## Author Contributions

JR and MA analyzed the data and drafted the first version of the manuscript; JB, AD, CD, and WS contributed data and assisted in data analysis and interpretation and in the drafting of the manuscript. TB conceived the study, contributed to data analysis, and drafted the first version of the manuscript. JR, MA and TB designed the app and JR programmed the app. All authors contributed to manuscript revision, read, and approved the submitted version.

## Funding

This work was supported by the Gates Foundation (INV-002098). JB received support from the UK Medical Research Council (MRC) and the UK Department for International Development (DFID; MR/K012126/1) under the MRC–DFID Concordat agreement and as part of the EDCTP2 program supported by the EU. TB is supported by a European Research Council Consolidator Grant (ERC-CoG 864180; QUANTUM). TB and MA are further supported by an AMMODO Science Award (2019).

## Conflict of Interest

The authors declare that the research was conducted in the absence of any commercial or financial relationships that could be construed as a potential conflict of interest.

## Publisher’s Note

All claims expressed in this article are solely those of the authors and do not necessarily represent those of their affiliated organizations, or those of the publisher, the editors and the reviewers. Any product that may be evaluated in this article, or claim that may be made by its manufacturer, is not guaranteed or endorsed by the publisher.
