# Supplementary material for: Quantifying Reductions in Plasmodium falciparum Infectivity to Mosquitos: A Sample Size Calculator to Inform Clinical Trials on Transmission-Reducing Interventions
Source: Front Immunol. 2022 Jun 3;13:899615. doi: 10.3389/fimmu.2022.899615 (PMC9205189; doi:10.3389/fimmu.2022.899615)
Supplement: Supplementary file 1 [file DataSheet_1.docx]

Supplementary material

# Table of contents

Supplementary table 1 2

Supplementary methods 3

References 7

**Supplementary table 1 – Reference values form multiple transmission blocking intervention datasets**

|  |  | **Baseline %infected mosquitos** | **ICC** | **SD of the random intercept** | **Dispersion parameter** | **Baseline oocyst density** |
| --- | --- | --- | --- | --- | --- | --- |
| **DMFA** |  |  |  |  |  |  |
|  | 2014 | 17.02 | 0.52 | 1.871 | - | - |
|  | 2016 | 14.21 | 0.64 | 2.429 | - | - |
|  | 2019 | 15.28 | 0.65 | 2.449 | - | - |
|  | 2020 | 14.57 | 0.63 | 2.382 | - | - |
| **SMFA** |  |  |  |  |  |  |
|  | 1 | - | 0.00 | 0.003 | 5.740 | 23.40 |
|  | 2 | - | 0.00 | 0.001 | 4.920 | 26.47 |
|  | 3 | - | 0.30 | 0.286 | 5.302 | 43.60 |
|  | 4 | - | 0.00 | 0.003 | 5.385 | 23.95 |
|  | 5 | - | 0.00 | 0.003 | 3.792 | 16.12 |
|  | 6 | - | 0.50 | 0.429 | 6.031 | 24.67 |
|  | 7 | - | 0.35 | 0.392 | 3.509 | 20.98 |
|  | 8 | - | 0.24 | 0.453 | 1.276 | 6.10 |
|  | 9 | - | 0.67 | 0.654 | 5.233 | 17.36 |
|  | 10 | - | 0.28 | 0.389 | 2.522 | 10.85 |
|  | 11 | - | 0.13 | 0.219 | 3.810 | 9.87 |

# Supplementary methods

## Transmission reducing activity

### The model

We estimate the transmission reducing activity by assuming that oocyst density follows a negative binomial distribution (1-3). The logarithm of the mean/expected number of oocysts in the j^th^ mosquito fed on subject i is:

$\text{log}\left( \mu_{ij} \right)=\beta_{0}+\beta_{1}x_{ij}+z_{i},$

where $x_{ij}$ is an indicator variable taking the values 0 and 1 for a pre- and a post-intervention measurement respectively of the log oocyst count $y_{ij}$ for each mosquito $j$ clustered within participant $i$. $\beta_{0}$ and $\beta_{1}$ are regression coefficients to be estimated. $z_{i}$ are random intercepts that are used to account for the correlation from mosquitos who were fed blood from the same participant. The $z_{i}$’s are assumed to be normally distributed with variance $\sigma^{2}$.

Larger random effects variances imply a larger degree of within cluster correlation while $\sigma^{2}=0$ implies that the data are independent. Negative binomial regression models, unlike Poisson regression models, do not assume that the variance is equal to the mean. Instead variance is estimated as a function of the mean and a dispersion parameter $\theta$ such that $var\left( y \right)=\mu+\frac{\mu^{2}}{\theta}$ where $E\left( y \right)=\mu$ (4). When $\theta\to\infty$, the variance approaches the value of the mean, and the negative binomial model converges to a Poisson regression model. Together, $\sigma^{2}$ and $\theta$ can be used to estimate the intra-cluster correlation (ICC) for a mixed effects negative binomial regression model (5) as

$$ICC=\frac{\sigma^{2}}{\sigma^{2}+\text{log}\left( 1+\frac{1}{\theta}+\frac{1}{\text{exp}(\beta_{0}+0.5\sigma^{2})} \right)}$$

where $\beta_{0}$ is an estimate from an intercept-only model, which could be thought of as the expected log oocyst count for a pre-intervention measurement.

TRA is equivalent to calculating $\left( 1-RR \right)\times100$ where $RR=exp(\beta_{1})$ is the risk ratio. Thus,

$$TRA=\left( 1-\text{exp}\left( \hat{\beta}_{1} \right) \right)\times100.$$

### The simulation algorithm

1. We generate $n$ participants.
2. For each participant we generate $m$ mosquito dissections pre-intervention (baseline) and $m$ mosquito dissections post-intervention with a variable $x_{ij}$ to indicate whether the mosquito dissection for mosquito $j$ from participant $i$ is pre-intervention $(x_{ij}=0)$ or post-intervention $(x_{ij}=1)$.
3. We simulate individual-specific random intercepts $(z_{i})$ from a normal distribution with a mean of 0 and a specified standard deviation $\sigma$, i.e. $Z\sim N(0,\sigma)$.
4. Lastly we generate the oocyst counts $(y_{ij})$ for each participant $i$ and mosquito $j$ using a negative binomial distribution such that $Y\sim NB(\mu,\theta)$ where $\theta$ is the anticipated dispersion parameter and $\mu_{ij}=\text{exp}(\beta_{0}+\beta_{1}x_{ij}+z_{i})$ where $\beta_{0}$ is the anticipated mean of the log oocyst counts at baseline and $\beta_{1}$ is the anticipated regression coefficient which is a function of the anticipated TRA, such that

$$\beta_{1}=\text{log}\left( 1-\frac{TRA}{100} \right).$$

### The statistical test

The general expectation is that $TRA>0$, however there may be situations where we want to perform a one-sided test

$$H_{0}:TRA\leq\tau$$

$$H_{1}:TRA>\tau$$

where $\tau$ is a defined % threshold that we wish to show our TRA estimate exceed or power our study to detect TRA above this threshold value. To do so, we note that the test is equivalent to testing based on the parameter $\beta_{1}$. i.e.

$$H_{0}: \beta_{1}\geq\log\left( 1-\frac{\tau}{100} \right)$$

$$H_{0}: \beta_{1}<\log\left( 1-\frac{\tau}{100} \right).$$

We obtain the standard errors for the $\beta_{1}$ from the model, and then compute $p$ (the p-value) of the test above. We repeat this for each simulated dataset and the empirical power is calculated as the percentage of $p$’s either less than 0.05 or less than 0.025, depending on the preferred level of significance $\alpha$. It is also worth noting that $\alpha=0.025$ is preferred in one sided tests.

## Transmission blocking activity

### The model

When estimating transmission-blocking activity, the oocyst density distribution is not important but rather the percentage mosquitos that has at least one oocyst: the proportion infected mosquitos. We thus estimate the transmission blocking activity by using a logistic regression model to model the probability of oocyst presence (6, 7) through

$$\text{log}\left( \frac{P(y_{ij}\geq1)}{P(y_{ij}=0)} \right)=\beta_{0}+\beta_{1}x_{ij}+z_{i},$$

where $x_{ij}$ is an indicator variable taking the values 0 and 1 to indicate a pre- or post-intervention measurement respectively, and $y_{ij}$ is the number of oocysts for an individual mosquito $j$ that fed on a sample of participant $i$ such that $y_{ij}\geq1$ indicates the presence of oocysts and $y_{ij}=0$ indicates the absence of oocysts. Regression coefficients $\beta_{0}$ (intercept) and $\beta_{1}$ (effect size of the intervention) are to be estimated. Here too, the $z_{i}$ are normally distributed random effects with mean 0 and variance $\sigma^{2}$ to be estimated.

In mixed effects logistic regression, unlike in mixed effects linear regression or mixed effects negative binomial regression, the interpretation of the estimated $\beta^{'}s$ are conditional on the random intercepts (i.e. a participant-specific interpretation) and they do not have a marginal (population-level) interpretation (8, 9). This means that conversion of the estimated coefficients into the baseline proportion infected mosquitos and the TBA are interpreted as effects for the average participant and not the population average. We use an approximation, outlined below, to transform the estimated coefficients from a participant-specific interpretation to a population-average interpretation (9).

In the model $\beta_{0}$ is the anticipated mean of the log odds pre-intervention $(x_{ij}=0)$ for the average participant $(z_{i}=0)$. Now $\beta_{1}$ is the individual-specific anticipated difference in log odds post-intervention vs pre-intervention such that $OR=\text{exp}\left( \beta_{1} \right)$ is the odds ratio. We can then approximate population averaged effects $\beta_{m0}$ and $\beta_{m1}$ through $\beta_{mk}=\frac{\beta_{k}}{\sqrt{c^{2}\sigma^{2}+1}}$ where $c=\frac{16\sqrt{3}}{15\pi}$ and $\sigma^{2}$ is the random effects variance (10).

The population averaged proportion of infected mosquitos pre-intervention can then be estimated as $p_{0}=\frac{\exp\left( \beta_{m0} \right)}{1+\exp\left( \beta_{m0} \right)}$. TBA is defined as the population average % reduction in the proportion of infected mosquitos post-intervention ($p_{1}$) relative to pre-intervention $\left( p_{0} \right)$, i.e.

$$TBA=\left( \frac{p_{0}-p_{1}}{p_{0}} \right)\times100=(1-RR)\times100$$

where $RR$ is the risk ratio. In a logistic regression model the odds ratio is directly estimated i.e. through $\exp(\beta_{m1})$, but the risk ratio is dependent on the odds ratio and the pre-intervention proportion of infected mosquitos, i.e.

$$RR=\frac{\exp(\beta_{m1})}{\left( \exp\left( \beta_{m1} \right)-1 \right)\times p_{0}+1}.$$

Lastly, the ICC can be estimated from the random effects variance through

$$ICC=\frac{\sigma^{2}}{\left( \frac{\pi^{2}}{3}+\sigma^{2} \right)}.$$

### The simulation algorithm

1. We generate $n$ participants.
2. For each participant we generate $m$ mosquito dissections pre-intervention (baseline) and $m$ mosquito dissections post-intervention with a variable $x_{ij}$ to indicate whether the mosquito dissection for mosquito $j$ from participant $i$ is pre- $(x_{ij}=0)$ or post-intervention $(x_{ij}=1)$.
3. We simulate individual-specific random intercepts $(z_{i})$ from a normal distribution with a mean of 0 and a specified standard deviation $\sigma$, i.e. $Z\sim N(0,\sigma)$. This standard deviation of the random effects can be estimated for a given intra-cluster correlation (ICC) through

$$\sigma=\sqrt{\frac{\pi^{2}\times ICC}{3\times(1-ICC)}}.$$

4. More specifically for step 5 below, it is not as meaningful to specify the individual-specific effects $\beta_{0}$ and $\beta_{1}$ without first anticipating the population-level effects for which we want to generate the simulated data. Instead we specify the population average baseline proportion infected mosquitos, $p_{0}$, and the population average TBA and calculate the population-level effects $\beta_{m0}=\text{log}\left( \frac{p_{0}}{1-p_{0}} \right)$ and $\beta_{m1}=\text{log}\left( 1-\frac{TBA}{100-100\times p_{0}+TBA\times p_{0}} \right)$. Then through the approximation equations $\beta_{k}=\beta_{mk}\sqrt{c^{2}\sigma^{2}+1}$ where $c=\frac{16\sqrt{3}}{15\pi}$ and $\sigma^{2}$ is the random effects variance (10), we determine the individual-specific effects $\beta_{0}$ and $\beta_{1}$ to be used in step 5.
5. Lastly we generate a binary outcome for infection status

$$y_{ij}=\left\{ \begin{aligned} 1 \left( \text{infected} \right)\text{ } \\ 0 (\text{uninfected}) \end{aligned} \right.$$

for each mosquito $j$ for participant $i$ using a Bernoulli distribution such that $Y\sim Bernoulli(p_{ij})$ where $p_{ij}=P(y_{ij}=1)$ and calculated through a logistic regression model $\log\left( \frac{p_{ij}}{1-p_{ij}} \right)=\beta_{0}+\beta_{1}x_{ij}+z_{i}$ with specified parameters $\beta_{0}$ and $\beta_{1}$ and simulated $z_{i}$ as defined in the step above where $\beta_{0}$ and $\beta_{1}$ are parameters from a individual-specific model.

### The statistical test

We want to perform a one-sided test

$$H_{0}:TBA\leq\tau$$

$$H_{1}:TBA>\tau$$

where $\tau$ is some % threshold that we wish to show our TBA exceeds or power our study to detect TBA above this threshold. To do so, we note that the test is equivalent to testing based on the risk ratio $RR$. i.e.

$$H_{0}:\log RR\geq\log\left( 1-\frac{\tau}{100} \right)$$

$$H_{0}: \log RR<\log\left( 1-\frac{\tau}{100} \right).$$

We obtain the standard errors for the $\log RR$ using the delta method (11, 12), and then compute $p$ (the p-value) of the test above. We repeat this for each simulated dataset and the empirical power is calculated as the percentage of $p$’s either less than 0.05 or less than 0.025, depending on the preferred level of significance $\alpha$. It is also worth noting again that $\alpha=0.025$ is preferred in one sided tests.

# References

1. Blagborough AM, Churcher TS, Upton LM, Ghani AC, Gething PW, Sinden RE. Transmission-Blocking Interventions Eliminate Malaria from Laboratory Populations. *Nature communications* (2013) 4(1):1812. doi: 10.1038/ncomms2840.

2. Medley GF, Sinden RE, Fleck S, Billingsley PF, Tirawanchai N, Rodriguez MH. Heterogeneity in Patterns of Malarial Oocyst Infections in the Mosquito Vector. *Parasitology* (1993) 106 ( Pt 5):441-9. Epub 1993/06/01. doi: 10.1017/s0031182000076721.

3. Billingsley PF, Medley GF, Charlwood D, Sinden RE. Relationship between Prevalence and Intensity of Plasmodium Falciparum Infection in Natural Populations of Anopheles Mosquitoes. *The American journal of tropical medicine and hygiene* (1994) 51(3):260-70. Epub 1994/09/01. doi: 10.4269/ajtmh.1994.51.260.

4. Venables WN, Ripley BD. *Modern Applied Statistics with S*. 4 ed: Springer, New York, NY (2002). XII, 498 p.

5. Nakagawa S, Johnson PCD, Schielzeth H. The Coefficient of Determination R(2) and Intra-Class Correlation Coefficient from Generalized Linear Mixed-Effects Models Revisited and Expanded. *J R Soc Interface* (2017) 14(134). Epub 2017/09/15. doi: 10.1098/rsif.2017.0213.

6. Churcher TS, Blagborough AM, Delves M, Ramakrishnan C, Kapulu MC, Williams AR, et al. Measuring the Blockade of Malaria Transmission--an Analysis of the Standard Membrane Feeding Assay. *Int J Parasitol* (2012) 42(11):1037-44. Epub 2012/10/02. doi: 10.1016/j.ijpara.2012.09.002.

7. Kapulu MC, Da DF, Miura K, Li Y, Blagborough AM, Churcher TS, et al. Comparative Assessment of Transmission-Blocking Vaccine Candidates against Plasmodium Falciparum. *Sci Rep* (2015) 5:11193. Epub 2015/06/13. doi: 10.1038/srep11193.

8. Muff S, Held L, Keller LF. Marginal or Conditional Regression Models for Correlated Non-Normal Data? *Methods in Ecology and Evolution* (2016) 7(12):1514-24. doi: <https://doi.org/10.1111/2041-210X.12623>.

9. Pavlou M, Ambler G, Seaman S, Omar RZ. A Note on Obtaining Correct Marginal Predictions from a Random Intercepts Model for Binary Outcomes. *BMC Medical Research Methodology* (2015) 15(1):59. doi: 10.1186/s12874-015-0046-6.

10. Zeger SL, Liang KY, Albert PS. Models for Longitudinal Data: A Generalized Estimating Equation Approach. *Biometrics* (1988) 44(4):1049-60. Epub 1988/12/01.

11. Doob JL. The Limiting Distributions of Certain Statistics. *The Annals of Mathematical Statistics* (1935) 6(3):160-9, 10.

12. Oehlert GW. A Note on the Delta Method. *The American Statistician* (1992) 46(1):27-9. doi: 10.2307/2684406.
